# Hierarchical structure of cascade of primary and secondary periodicities in Fourier power spectrum of alphoid higher order repeats

**DOI:** 10.1186/1471-2105-9-466

**Published:** 2008-11-03

**Authors:** Vladimir Paar, Nenad Pavin, Ivan Basar, Marija Rosandić, Matko Glunčić, Nils Paar

**Affiliations:** 1 Faculty of Science, University of Zagreb, Bijenička 32, 10000 Zagreb, Croatia; 2 Max Planck Institute for the Physics of Complex Systems, Noethnitzer Str. 38, 01187 Dresden, Germany; 3 Department of Internal Medicine, University Hospital Rebro, University of Zagreb, Kišpatićeva 12, 10000 Zagreb, Croatia

## Abstract

**Background:**

Identification of approximate tandem repeats is an important task of broad significance and still remains a challenging problem of computational genomics. Often there is no single best approach to periodicity detection and a combination of different methods may improve the prediction accuracy. Discrete Fourier transform (DFT) has been extensively used to study primary periodicities in DNA sequences. Here we investigate the application of DFT method to identify and study alphoid higher order repeats.

**Results:**

We used method based on DFT with mapping of symbolic into numerical sequence to identify and study alphoid higher order repeats (HOR). For HORs the power spectrum shows equidistant frequency pattern, with characteristic two-level hierarchical organization as signature of HOR. Our case study was the 16 mer HOR tandem in AC017075.8 from human chromosome 7. Very long array of equidistant peaks at multiple frequencies (more than a thousand higher harmonics) is based on fundamental frequency of 16 mer HOR. Pronounced subset of equidistant peaks is based on multiples of the fundamental HOR frequency (multiplication factor *n *for *n*mer) and higher harmonics. In general, *n*mer HOR-pattern contains equidistant secondary periodicity peaks, having a pronounced subset of equidistant primary periodicity peaks. This hierarchical pattern as signature for HOR detection is robust with respect to monomer insertions and deletions, random sequence insertions etc. For a monomeric alphoid sequence only primary periodicity peaks are present. The 1/*f*^*β *^– noise and periodicity three pattern are missing from power spectra in alphoid regions, in accordance with expectations.

**Conclusion:**

DFT provides a robust detection method for higher order periodicity. Easily recognizable HOR power spectrum is characterized by hierarchical two-level equidistant pattern: higher harmonics of the fundamental HOR-frequency (secondary periodicity) and a subset of pronounced peaks corresponding to constituent monomers (primary periodicity). The number of lower frequency peaks (secondary periodicity) below the frequency of the first primary periodicity peak reveals the size of *n*mer HOR, i.e., the number *n *of monomers contained in consensus HOR.

## Background

### Introduction

Repeat sequences are a common feature of genomes [1-3]. The detection and study of periodicity in genomic sequences has been an area of increasing interest. Signal processing approaches to periodicity detection methods are attracting significant attention in genomic DNA investigations of approximate repeats because they are rather robust in the presence of substitutions, insertions and deletions and may identify approximate periodicities in DNA sequences. Different computational techniques have been used: Fourier spectral analysis [4-20], wavelet transform [21], DNA walk analysis [22-25], information theory measures [26-28], informational decomposition [29,30], quaternionic periodicity transform [31], exactly periodic subspace decomposition [32,33], portrait method [34], enhance algorithm for distance frequency distribution [35], etc.

### Discrete Fourier transformation (DFT) based methods

Spectral analysis employing Discrete Fourier transform is used to reveal periodicity in symbolic sequences, like genomic and protein sequences [7,9,14,16,17,20,36-53], to investigate long-range correlations [4,5,54,55] and to study the problem of sequence similarity [14,56-62].

### DFT identification of approximate repeats

A peak at a frequency *f *in Fourier power spectrum of base correlations of a given genomic sequence shows a kind of *l *= 1/*f *– base periodicity, exact or approximate [14-16,63]. In the ideal case of perfect periodicity, where a fragment of the length *l *is exactly repeated N times, periodicity generates a series of *l*-1 equidistant peaks in the power spectrum, at frequencies [14,16]:

*f*_1 _= 1/*l*, *f*_2 _= 2/*l*, *f*_3 _= 3/*l*, ... *f*_*l*-1 _= (*l*-1)/*l*

Approximate repeats, modified by random insertions and/or deletions with respect to perfect repeats, typical for genomic sequences of higher organisms, can often be identified using Fourier transform [14,16,17]. This procedure results in a characteristic system of equidistant peaks. However, it was noted that a disadvantage of methods based on Fourier transform may be that in cases of more pronounced deletions or insertions the periodicity cannot be detected, while deletions and insertions are frequent mutational events in genomic sequences [29,50].

### DFT identification of period three hidden periodicity

A sharp peak of period three was found in a search for periodic regularities on a sample set of human exons [5,9,10,22,54,60,64]. The three-base periodicity in exons is caused by unbalanced nucleotide distributions in the three coding positions, while in intron sequences the nucleotides distribute uniformly. The relative height of the corresponding peak in Fourier spectrum is a good discriminator of coding potential and has been used to detect coding regions [9,14,37,45,49,65-75].

### DFT identification of long-range correlations

Statistical studies of DNA sequences have been instigated by finding of the 1/*f*^*β *^long-range power-law correlations in human genomic sequences, indicating the presence of scale invariant structure [4,5,22], implying that the underlying system shows fractal properties [25,76,77]. The lack of long enough sequences and the use of different methods of estimating the correlations, leading to some results not strictly comparable to each other contributed to controversies regarding findings on long-range correlations, like the presence of these correlations only in non-coding or in all human genomic sequences, and their presence in other organisms [5,6,23,36,78-83]. Non-stationary analysis of DNA sequences has shown that both coding and non-coding sequences exhibit long-range correlations, with the average spectral exponent of non-coding segments being higher than its counterpart for coding segments [84]. With the availability of large sequences and extended statistical computations, showing power-law correlations over four or five orders of magnitude, with exponents which are consistent with previous results obtained analyzing short sequences, such correlations in human DNA, with fractal-like scaling, are now commonly accepted [27,28,45]. It has been pointed out that the mosaic structure of genome is presumably responsible for long-range correlations [79,85,86]. At very low frequencies (for example, *f *< 10^-6^) the power spectrum flattens out [87-89]. It should be noted that the attribution of long-rang correlations exclusively to large-scale variations of nucleotide density responsible for 1/*f*^*β *^spectra is not quite correct. Generally, even large-scale variations of nucleotide density may produce patterns different from 1/*f*^*β *^spectra.

### DFT identification of alphoid higher order repeats (HOR)

Here we investigate the application of Fourier analysis to human alpha satellite tandem repeats and the associated higher order repeats (HORs). Alphoid arrays consist of tandem repeats of alpha satellite monomer unit of approximately 171 bp, which form chromosome-specific higher order repeats (HOR) or monomeric organization consisting of diverged monomers [90-104]. Alpha satellite monomers within HOR exhibit substantial mutual sequence divergence (20–40%), while HORs exhibit much lower mutual divergence (< 5%) [98]. Such a case is interesting for Fourier analysis because it has a two-level hierarchy of approximate homology.

Alpha satellite DNA is characterized by many levels of hierarchical organization in genomes, from suprachromosomal families to chromosome-specific subsets, to polymorphic variation within these subsets [90-103]. The higher order repeat organization is consistent with linear sets of diverged monomers becoming the unit of crossing-over during the process of sequence homogenization. The HOR units of alpha satellite monomers are organized in largely chromosome specific manner. The centromere of each human chromosome is characterized by one or more subsets of distinct alpha satellite HOR units. Analyses have revealed the presence of up to several thousand repeat units arranged in an apparently uninterrupted fashion in the centromere and forming arrays of several million base pairs. Alpha satellite HORs have been studied using restriction enzymes that cut higher order repeat unit [98,101]. Recently, HORs and monomeric alpha satellites have been studied by computational analysis of genomic sequences from the NCBI genome assembly [104-108].

As a case study we consider a 16 mer HOR at the loci D7Z2 and D7Z1 in human chromosome 7 [94-97]. In [104] 16 mers were identified by DOTTER analysis; the presence of 16 mer was reported, but detailed HOR structure was not presented. In detailed computational studies of genomic sequence of the 193277-bp clone AC017075.8 (contig NT_023603.5), the 46 complete and 14 incomplete copies of 16 mer alphoid HOR were identified in the central domain (positions 31338 to 177434, total length 148147 bp) [105-107]. Preliminary study of power spectra discussed the general pattern and the signal-to-noise ratio [17]. These HOR copies are highly homologous (divergence from consensus less than 0.6% on the average), while divergence among monomers within each HOR copy is sizeable (20% on the average). (In accordance with common practice, monomer deletions or insertions, which appear in some HOR copies, are not taken into account in calculating divergence among HOR copies.) Such a long genomic sequence enables a highly precise determination of higher order periodicities. In the front domain of genomic sequence (31337 bp) and in the back domain (15843 bp), 199 alpha satellite monomers are present which are not organized into HORs and therefore are all mutually divergent by 20% or more. Only 29% of this bordering domain is not of alpha satellite type.

Our goal is to investigate the periodicities in the short-, medium-, and long-range order, related both to less homologous alphoid monomeric pattern and to more homologous alphoid higher-order repeats and to correlate the two levels of periodicity, primary (basic monomer periodicity) and secondary (HOR periodicity).

## Results and discussion

### DFT identification of HOR in AC017075.8 based on quartic mapping

The genomic sequence AC017075.8 (193277 bp) from chromosome 7 was transformed into numerical sequence using quartic mapping (Eq. 5) with parameters (Eq. 6) (see section Methods). The AC017075.8 sequence is used as a case study for the use of DFT method for interplay of monomeric and HOR repeats. In general, regions containing higher order repeat sequences can be located through the sliding window analysis, similarly as used in [16] for primary periodicity sequences. Analyzing complete nucleotide sequence we found domains having different repeat pattern, the central HOR domain and the bordering domains (front and back domains), in accordance with identifications obtained using other methods [94-97,105-108].

**Figure 1:**
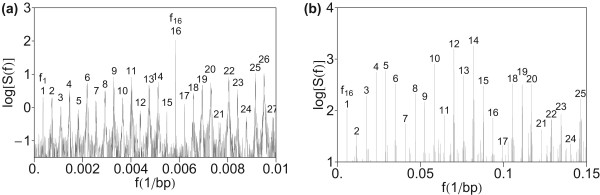
**Power spectrum of HOR domain in AC017075.8 computed by using quartic mapping (Eq. 6)**. (a) Low-frequency section *f *< 0.01 bp^-1^. Equidistant peaks above the background of noise are assigned consecutively by integers 1, 2, 3, ... The fundamental frequency of peak no. 1 is denoted by *f*_1_. Frequencies of the peaks no. 2, 3, ... are denoted by *f*_2 _= 2·*f*_1_, *f*_3 _= 3·*f*_1_, ..., respectively. (b) Medium frequency section up to *f *= 0.15 bp^-1^. Pronounced equidistant peaks above the background of noise are assigned consecutively by integers 1, 2, 3, ... The frequency of monomeric peak no. 1 is the HOR peak at frequency *f*_16 _= 16·*f*_1 _from Figure 1a). Frequencies of monomeric peaks no. 2, 3, ... are 2·*f*_16 _= 2·16*f*_1_, 3·*f*_16 _= 3·16*f*_1_, ..., respectively (in terms of fundamental HOR frequency *f*_1 _from Figure 1a). The noise level is such that the monomeric peaks at frequencies *nf*_16 _are clearly seen above the background.

The low-frequency part *f *< 0.01 bp^-1 ^of the power spectrum of HOR domain in AC017075.8 is displayed in Figure 1a) and the power spectrum up to the frequency 0.15 bp^-1 ^in Figure 1b). The computed power spectrum at low frequencies shows equidistant peaks at frequencies

*f*_*n *_= *n*·*f*_1_,   n = 1, 2, 3, ...

The fundamental frequency *f*_1 _corresponds to the 2734-bp HOR. (Due to truncation of data set and the associated precision limit of 7.6·10^-6^, a more precise value can be deduced from the systematic of higher multiples).

These equidistant peaks are identified over a very broad interval, up to n ≈ 1000. In fact, all prominent peaks above the white noise background in the power spectrum are multiples of the fundamental frequency *f*_1_. We note that such an extremely regular pattern can be rarely found even in the most regular dynamical systems in physics and engineering.

In addition to the standard spectral density S_*f *_(*f*_*n*_) at frequency *f*_*n *_(square of absolute value of Fourier amplitude), we define an effective spectral density

$$S_{eff}(f_{n})=S_{f}(f_{n})\frac{f_{1}}{f_{n}},$$

renormalized in order to increase the relative weight of low-frequency with respect to high frequency peaks. The effective values *S*_*eff *_corresponding to frequencies *f*_1_, *f*_2 _= 2*f*_1_, *f*_6 _= 6*f*_1_, and *f*_16 _= 16*f*_1 _are 2.025, 0.973, 0.895, and 6.584, respectively.

The prominent peak at the frequency *f*_16 _= 0.005852 bp^-1 ^corresponds to approximately 171 bp length. More precisely, 1/*f*_16 _= 170.88 bp. It corresponds to a set of alpha satellite monomers which constitute consensus HOR (nine 171-bp, five 170-bp, one 172-bp, and one 173-bp copy variants). Alternatively, the HOR period 1/*f*_1 _= 2734 bp could be also expressed as multiple of monomer period 1/*f*_16 _= 171 bp. The low-frequency peaks at *f*_1_, *f*_2_, ..., *f*_15 _are subharmonics of the monomer frequency *f*_16_.

In the frequency region above the monomer frequency *f*_16 _(Figure 1b), within the set of multiple frequencies *nf*_1 _(*n *> 16) we find a prominent subset of higher harmonics at frequencies that are multiples of the monomer frequency *f*_16 _: 2*f*_16 _, 3*f*_16_, 4*f*_16_, ... This subset with band head at the frequency *f*_16 _will be referred to as monomeric band.

Fourier analysis works well enough for studying relatively short periodicities while the statistical significance of longer periodicities will be decreased by the presence of shorter periodicities [29]. Thus, the statistical significance of longer periods was predicted to be a sort of smeared through statistical significance of shorter periods, i.e., for harmonics with longer periods the damping effect may be more pronounced [29]. We show here that the DFT method is applicable to alphoid HORs up to very long periodicities (up to several kilobases).

Generally, the fundamental frequency for equidistant pattern in the power spectrum corresponds to the periodicity of highest order in a given sequence, i.e., to the period of HOR (secondary periodicity). Specifically, in the HOR domain of AC017075.8 the fundamental frequency in power spectrum corresponds to the period of HOR consensus unit, 2734 bp.

Although the HOR copies are much more homologous to each other than the constituent alpha satellite monomers among themselves, the number of monomers corresponding to primary periodicity (at frequencies *f*_16_, 2*f*_16_, 3*f*_16_, ...) is much higher than the number of HOR copies corresponding to secondary periodicity (at frequencies *f*_1_, *f*_2_, *f*_3_, ...), and therefore the peaks of primary periodicity have higher spectral strengths.

### Robustness of DFT results for hierarchical structure of cascade of primary and secondary periodicities using different genomic into numerical sequence mapping

The difficulty with DFT approach may be dependence on a particular labeling adopted. For example, some of the relevant harmonic structure can be hidden (or exposed) by the symbolic-to-numeric mapping [111]. To check the required mapping invariance, we investigate whether the hierarchical periodic pattern shown in this paper is robust with respect to a particular choice of procedure for transforming symbolic to numerical sequence.

#### Test computation for quartic mapping deduced from systematic of purine/pyrimidine and strong/weak bond characteristics

**Figure 2:**
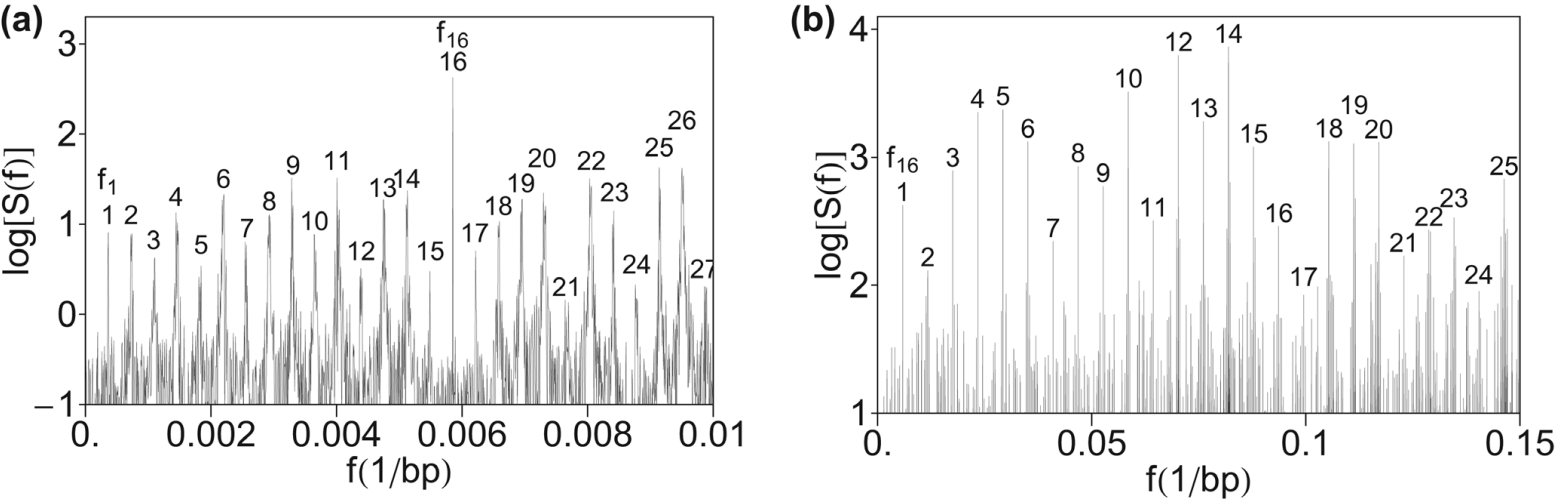
**Power spectrum of HOR domain in AC017075.8 computed by using quartic mapping with parameters (Eq. 8)**. (a) Low-frequency section. (b) Medium frequency section.

To test robustness of hierarchical structure obtained in Figures 1a) and 1b), we have first computed the power spectrum of HOR domain in AC017075.8, using the quartic mapping (Eq. 5) with parameters (Eq. 8) (Figures 2a) and 2b)). The quartic parametrization (Eq. 8) was based on combined consideration of purine/pyrimidine and strong/weak characteristics of nucleotides [113]. Up to an overall normalization, this spectrum is practically identical as obtained in the computation in Figure 1. This is understandable because of linear relation between mapping parameters (Eq. 6) and (Eq. 8).

#### Test computation for quartic mapping deduced from reduced dimensionality of frequency spectrum in symmetric manner

**Figure 3:**
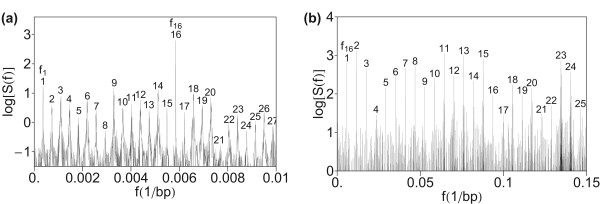
**Power spectrum of HOR domain in AC017075.8 computed by using quartic mapping and reduced dimensionality with parameters (Eq. 9)–(Eq. 11)**. (a) Low-frequency section. (b) Medium frequency section.

A further test was performed using quartic mapping (Eq. 5) with reduced dimensionality of the frequency spectrum representation from four to three with parameters (Eq. 9)–(Eq. 11) from [37]. The computed spectrum in Figures 3a) and 3b) shows a similar pattern of hierarchy of primary and secondary periodicity peaks as in Figure 1, confirming robustness of the method.

#### Test computation by summing the squares of Fourier transforms of indicator sequences

**Figure 4:**
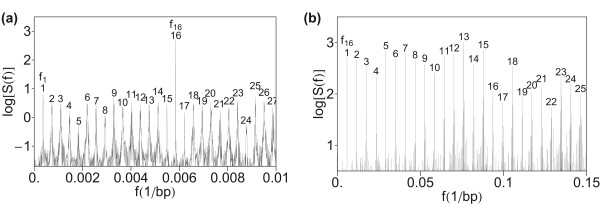
**Power spectrum of HOR domain in AC017075.8 computed by using total power spectrum obtained by summing squares of Fourier transform of indicator sequences (Eq. 4)**. (a) Low-frequency section. (b) Medium frequency section.

Finally, we test the robustness of hierarchical primary and secondary periodicity pattern by computing total power spectrum obtained by summing squares of Fourier transform of indicator sequences (Eq. 4). The resulting power spectrum in Figure 4 shows a similar hierarchical pattern as in Figure 1, confirming robustness of the method.

#### Hierarchical primary and secondary periodicity pattern in perfect HOR sequence

**Figure 5:**
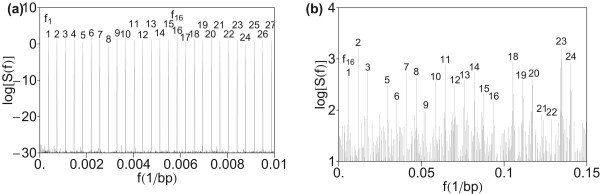
**Power spectrum of artificially constructed perfect HOR sequence formed by a sequence of 54 exactly identical HOR copies, equal to the 2734-bp consensus HOR corresponding to AC017075.8 in chromosome 7**. Computation is performed using quartic mapping with parametrization (Eq. 6). (a) Low-frequency section. (b) Medium frequency section.

The HOR sequence from AC017075.8 in chromosome 7, studied in Figures 1, 2, 3, 4, is characterized by a low divergence among 54 HOR copies in the sequence of only a few percent [105-108]. Here we construct an exact HOR sequence, with divergence among copies equal to zero, by forming a sequence of 54 identical HOR copies, equal to the 2734-bp consensus HOR corresponding to AC017075.8 in chromosome 7 [108]. The resulting power spectrum (Figure 5) shows a much more pronounced hierarchical secondary periodicity pattern than obtained for realistic HOR sequence in Figure 1. In this way analysis was extended to give some feel of how this perfect case appears when the periodicity is disrupted for a realistic genomic sequence.

### Robustness of power spectrum for hierarchical structure of cascade of primary and secondary periodicities for imperfect HORs

**Figure 6:**
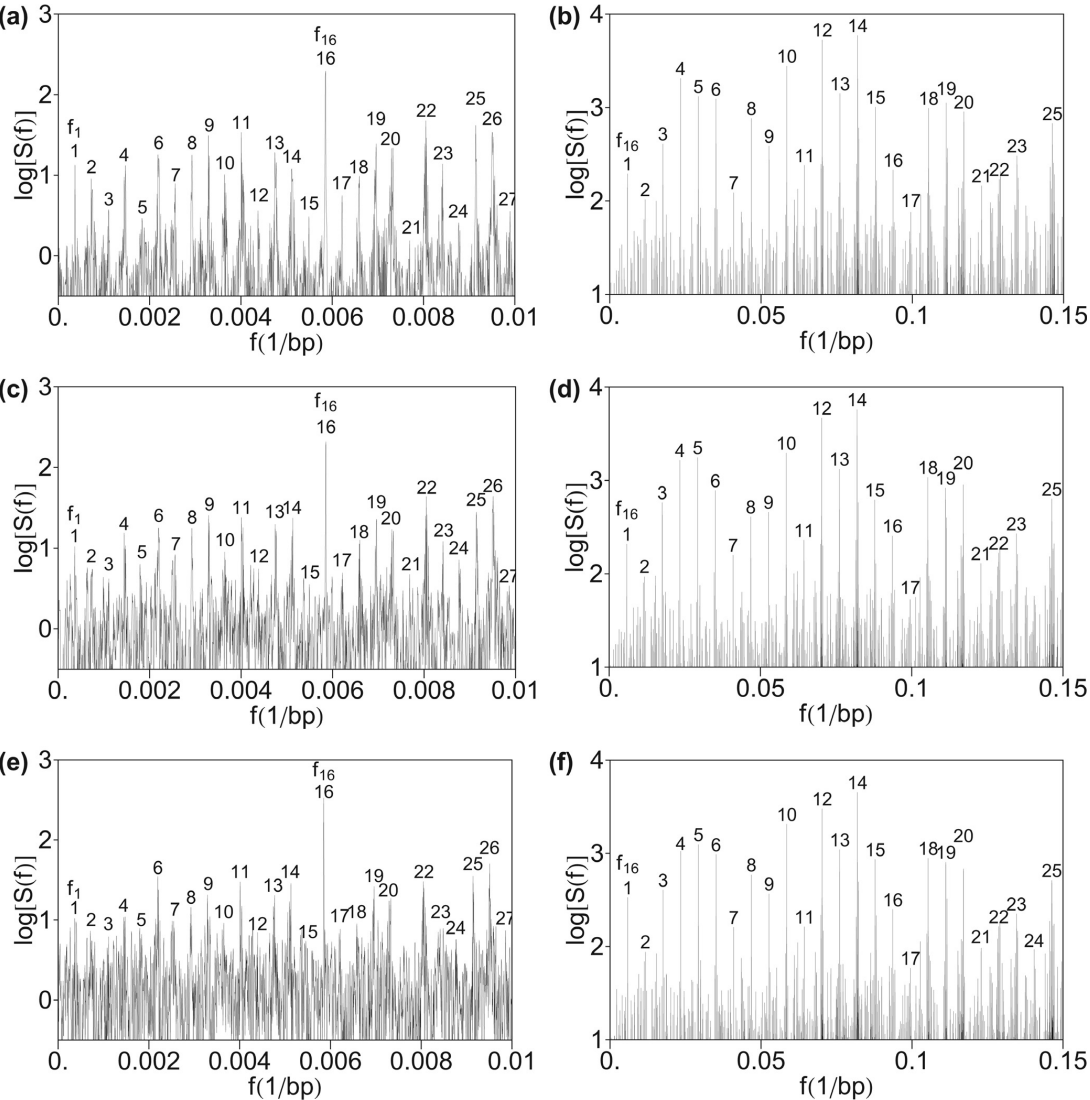
**Power spectrum of HOR domain in AC017075.8 with an additional insertion (random sequence obtained by random number generator) computed by DFT using quartic mapping with parameters (Eq. 8)**. (a) 10000-bp insertion; low-frequency section of the power spectrum. (b) 10000-bp insertion; medium-frequency section of the power spectrum. (c) 30000-bp insertion; low-frequency section of the power spectrum. (d) 30000-bp insertion; medium-frequency section of the power spectrum. (e) 60000-bp insertion; low-frequency section of the power spectrum. (f) 60000-bp insertion; medium-frequency section of the power spectrum. Insertions are placed at the location 65537 in AC017075.8.

In the next step we have investigated the robustness of the hierarchical periodicity pattern with an increase of imperfection in the HOR sequence. This is shown by random insertion of increased length inserted into the HOR sequence AC017075.8. Power spectra are presented for: 10000-bp random insertion (Figure 6a) and 6b)), 30000-bp random insertion (Figure 6c) and 6d)), and 60000-bp random insertion (Figure 6e) and 6f)). It is seen that the level of noise increases with increase of insertion length, but even in the case of 60000-bp random insertion (which is 40% of the total length of HOR copies) the hierarchical structure of primary and secondary periodicity can be identified (Figures 6e) and 6f)).

#### Noisy power spectrum of a random artificial sequence

**Figure 7:**
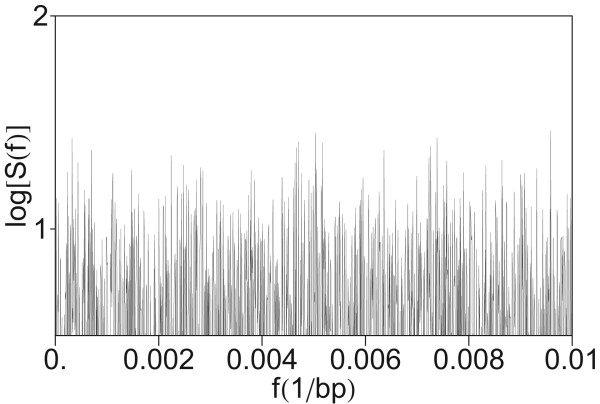
**Power spectrum of artificial random sequence constructed using random number generator**. Computation was performed using quartic mapping with parametrization (Eq. 8).

In order to test that the hierarchical primary and secondary periodicity pattern is not a numerical artifact, we have computed the power spectrum corresponding to a random sequence generated by random number generator, having the same length as the HOR sequence in AC017075.8 (148147 bp). Computation is performed using quartic mapping with paramerization (Eq. 8). From this power spectrum, shown in Figure 7, it is seen that the computational method does not generate hierarchical periodicity.

### Power spectrum pattern of monomeric alphoid domain in AC017075.8

**Figure 8:**
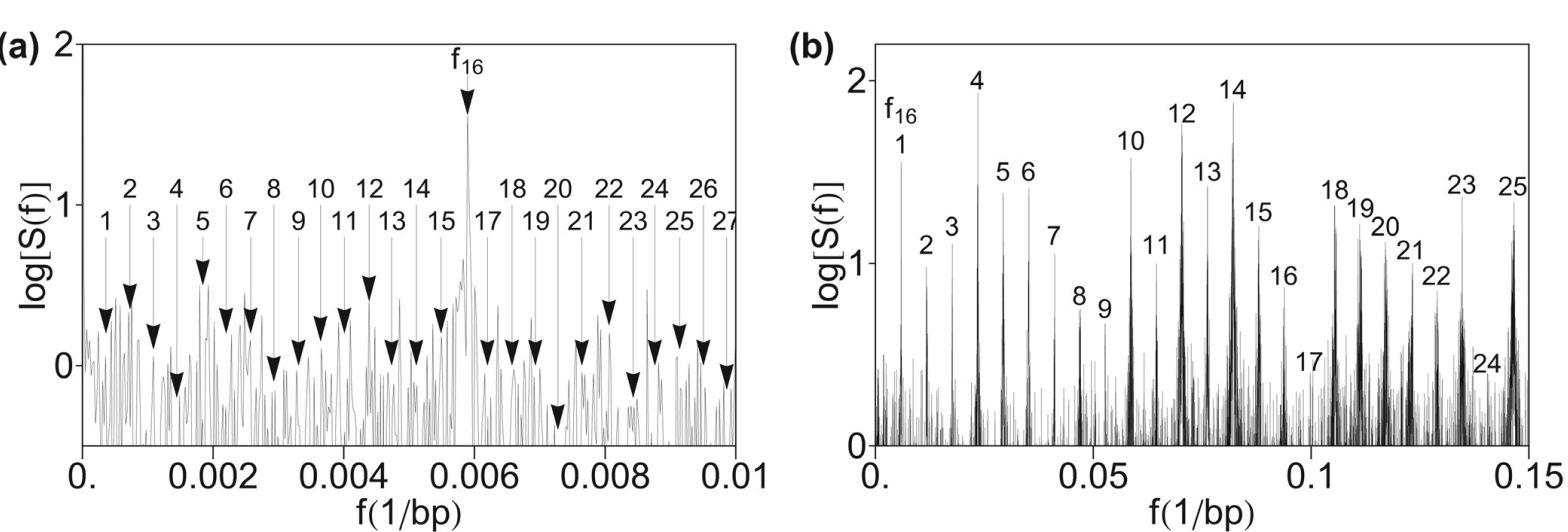
**Power spectrum of monomeric domains in AC017075.8 computed using quartic mapping with parametrization (Eq. 6)**. (a) Low-frequency section *f *< 0.01 bp^-1^. Positions corresponding to low-frequency peaks from Figure 1a), which are missing here, are indicated by arrows. (b) Medium frequency section up to *f *= 0.15 bp^-1^. Peaks are assigned in analogy to Figure 1b).

The low-frequency power spectrum (*f *< 0.01 bp^-1^) of combined front- and back-domains of AC017075.8 is displaced in Figure 8a) and the higher-frequency section in Figure 8b). A significant difference with respect to the central HOR domain is seen in the low-frequency region (Figure 8a): there are no prominent peaks below the frequency *f*_16 _(1/171 bp^-1^). In the front- and back-domains there is no peak corresponding to 16 mer HOR (at frequency *f*_1 _in Figure 1a), as well as to the multiples of *f*_1_: *f*_2 _= 2*f*_1_, *f*_3 _= 3*f*_1_, ..., *f*_15 _= 15*f*_1_, at frequencies below *f*_16 _which corresponds to the 171-bp monomer. In that case the frequency of the lowest peak in the power spectrum corresponds to the period 171 bp of consensus alpha monomer and the power spectrum contains only the monomeric band (primary periodicity). This reveals that HOR is absent in the front and back domains. A tandem of alpha satellite monomers, not organized into HORs, is referred to as monomeric [96,97]. As seen from our results, the noise is stronger in monomeric domains (front- and back-domains) than in the central HOR domain. On the other hand, the equidistant pattern associated with prominent peaks above the frequency *f*_16 _(Figure 8b), for monomeric domain is rather similar as for HOR domain (Figure 1b).

### Absence of low-frequency 1/*f*^*β*^-noise in DFT power spectrum

The 1/*f*^*β *^– noise is absent in the low-frequency region of power spectrum of AC017075.8, both in the central HOR domain (Figure 1a) and in the monomeric front- and back-domains (Figure 8a). This result is in accordance with expectations, because the sequence mainly consists of approximate repeats, without sizeable sequence-wide base composition fluctuations. Previously, some cases of absence of long range correlations in repeat sequences have been found. For example, in a sequence for beta globin on human chromosome 11 (HUMHBB, 73326 bp) two 6-kb segments without long-range correlations were identified, both including stretches of repetitive DNA [76].

**Figure 9:**
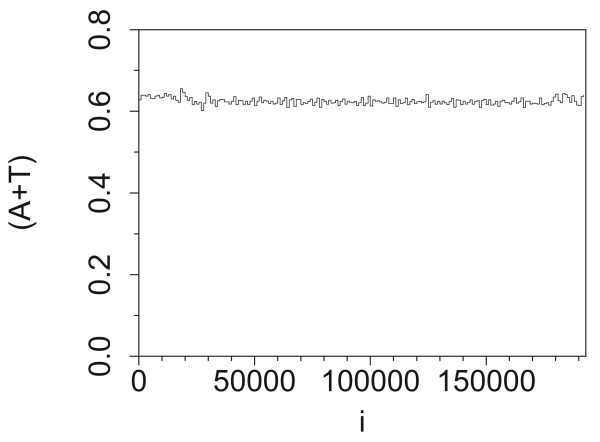
**Histogram of fraction of A+T nucleotides along the sequence AC017075.8 (bins of 1000 bp)**.

The A+T fraction in the AC017075.8 sequence is almost constant along the sequence (Figure 9). Using bins of 1 kb, the calculated fraction of A+T nucleotides is 0.625 ± 0.008%, with small fluctuations around the average value. This homogeneity of nucleotide density is in accordance with expectations that the 1/*f*^*β *^noise is related to varying ratio of pyrimidines and purines, or other nucleotide combinations, at base positions along DNA sequence [24,81].

**Figure 10:**
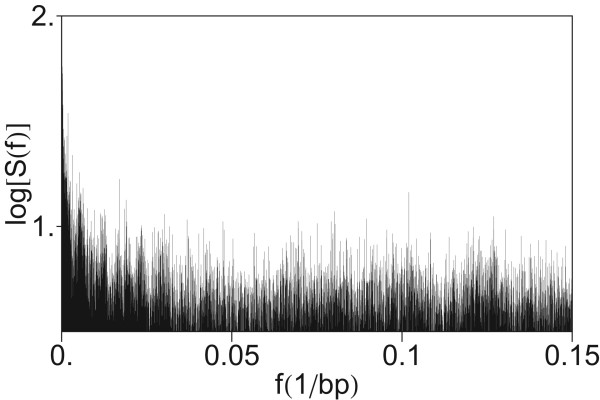
**Low frequency 1/*f*^*β *^– noise of the power spectrum of contig NT_004434.18 (1 Mb) in chromosome 1 outside of (peri)centromeric region**. Computation was performed using quartic mapping at parametrization (Eq. 7).

For comparison, to show that the DFT power spectrum method used here identifies the low-frequency 1/*f*^*β *^– noise, if present, we display the power spectrum computed for contig NT_004434.18 from chromosome 1 (Figure 10). This contig of about 1 Mb lies outside of (peri)centromeric region and is characterized by the presence of genes and absence of HORs. The power spectrum computed using quartic mapping at parametrization (Eq. 7) clearly shows the presence of low-frequency 1/*f*^*β *^– noise.

### Rank ordering for harmonics in alpha monomeric spectrum

In the power spectrum of our case study for genomic sequence in HOR domain the equidistant peaks corresponding to multiples of monomer frequency *f*_16 _are sizably stronger than the other peaks (Figure 1b). Among the low-frequency peaks in Figure 1b) the most pronounced peaks are 10*f*_16_, 12*f*_16_, and 14*f*_16_. The corresponding lengths are approximately 17 bp, 14 bp, and 12 bp, respectively. The 14-bp length may be related to the highest frequency of appearance of the 6-bp key string TTTTGA at the distance of 14 bp between two neighboring key strings. However, in general, the chosen mapping may influence the rank ordering of harmonics, as seen by comparing their relative heights in Figures 1, 2, 3, 4, 5. Thus, the effect of parameter choice for symbolic-to-numeric transformation may overshadow the effect of hidden genomic substructure.

### Absence of periodicity three in power spectrum of HORs

**Figure 11:**
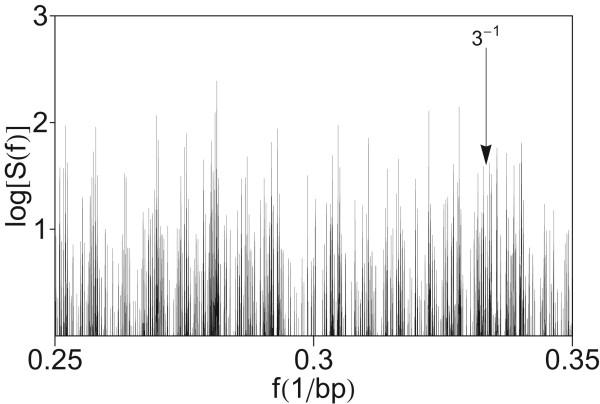
**Power spectrum of the HOR domain in AC017075.8 obtained by computation using quartic mapping from Figure 1 in higher frequency section (up to *f *= 0.35 bp-1)**.

**Figure 12:**
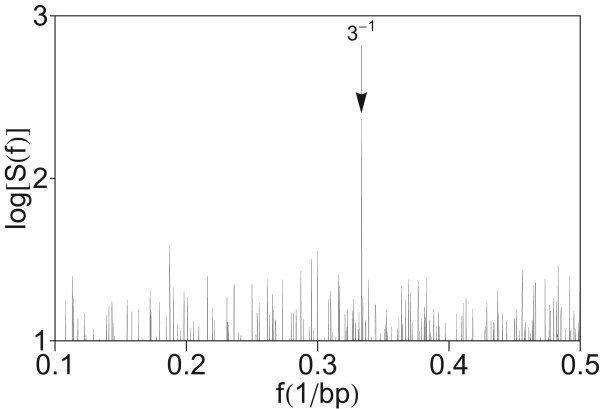
**Power spectrum of CDS from gene DNAH11 in AC004002.1 in chromosome 7 obtained by computation using quartic mapping (Eq. 8)**.

In previous investigations of Fourier power spectra of coding DNA sequences a major peak was found at the frequency *f *= 1/3 bp^-1^, related to the codon structure [5,14,37]. In the present case of a segment with entirely noncoding sequence, no peak appears at *f *= 1/3 bp^-1 ^(Figure 11). This is in accordance with previous conclusions that the period-3 feature is usually lacking or is weak in noncoding regions [7,9,37,39,41,66]. For comparison, using quartic mapping we computed the power spectrum of CDS from the gene DNAH11 in chromosome 7 (Figure 12). In this power spectrum obtained by computation using quartic mapping (Eq. 8), at the frequency *f *= 1/3 bp^-1 ^a pronounced peak is present.

### Use of power spectrum for identification of hierarchical primary and secondary periodicity pattern in chromosome 1

**Figure 13:**
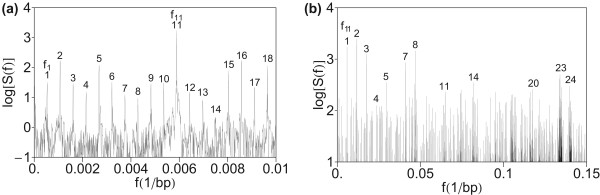
**Power spectrum of contig NT_077389.3 in chromosome 1, using quartic mapping with parametrization (Eq. 8)**. (a) Low-frequency section. (b) Medium frequency section.

The computation of power spectrum, shown here for the test case of 16 mer HOR in chromosome 7, can be extended for HOR identification and study in other chromosomes as well. As an example, we present in Figure 13 the power spectrum computed for contig NT_077389.3 in chromosome 1, using quartic mapping with parametrization (Eq. 8). Here we find a hierarchical pattern of primary and secondary periodicity (11 mers).

## Conclusion

We have demonstrated that DFT is a robust and efficient method to identify alphoid HORs in alpha satellite domain of genomic sequence. In the case of *n*mer HOR the lowest peak is at the fundamental frequency 1/(171n bp), which will be referred to as HOR-frequency. It is a head of band of equidistant peaks at frequencies equal to consecutive multiples of HOR-frequency, i.e., 1/(171n bp), 2/(171n bp), 3/(171n bp), ... This band is referred to as the HOR-band. Some peaks within the HOR-band form a strong-spectral-power subset with band head at monomer-frequency 1/(171 bp). This subset forms a band of equidistant peaks at frequencies which are multiples of monomer-frequency 1/(171 bp), i.e., it corresponds to the peaks at frequencies 1/(171 bp), 2/(171 bp), 3/(171 bp), .... This sub-band is referred to as the monomeric-band.

In the case of monomeric alpha satellites (not organized into HOR) the lowest peak is at the monomer-frequency 1/(171 bp). It is a head of monomeric-band built from peaks at frequencies 1/(171 bp), 2/(171 bp), 3/(171 bp), ....

DFT was applied here in the case study of genomic sequence AC017075.8 (193277 bp) from centromeric region in human chromosome 7. The central domain of AC017075.8 consists of 16-mer alphoid HOR copies. Thus the frequency of the lowest peak in the power spectrum (HOR-frequency) is 1/(171·16 bp). We identified in the power spectrum as many as one thousand peaks at frequencies equal to multiples of HOR-frequency, forming a HOR-band. Among these peaks in the HOR-band a subset of peaks at frequencies 1/(171 bp), 2/(171 bp), 3/(171 bp), .... is characterized by pronounced spectral power and represents the monomeric-band. This reveals hidden periodicities in the 171-bp monomer, i.e., a hierarchy of periodicities within the monomer sequence. Power spectra of both the HOR region and of the monomeric region show this pattern of hidden higher frequencies.

The case study shows that DFT is robust in detecting approximate HORs, even in the presence of substantial sequence insertions and deletions.

Additionally, the applicability of DFT method was shown for chromosome 1, where a hierarchical pattern of 11 mer HOR is present.

Computing DFT power spectra for anonymous genomic sequence using sliding windows for bins of about 50 kb and step size of about 10 kb provides an easily recognizable hierarchical two-level equidistant pattern in the power spectrum as signature of presence of HOR and gives a simple method to determine the size of HOR.

## Methods

### Discrete Fourier transform of genomic sequence

To apply DFT, one should first represent genomic sequence, a symbol sequence over the alphabet {*A*, *T*, *G*, *C*}, as a numerical sequence reflecting the characteristics of the symbol sequence. Several approaches have been used for solving the problem of transformation of a symbol sequence to numerical sequence.

A common mapping scheme is to decompose genomic sequence into four component indicator sequences. These binary indicator sequences, u_A(m)_, u_T(m)_, u_C(m)_, and u_G(m)_, take the value of either 1 or 0 at position m depending on whether the corresponding character is present or absent at that location, respectively. These indicator sequences were analyzed by respective Fourier transforms [5,9,39,40,55]. For pure DNA character strings (i.e., without assigning numerical values), to the binary indicator sequences u_A(m)_, u_T(m)_, u_C(m)_, and u_G(m) _correspond the DFT sequences

(3) u_A(k)_, u_T(k)_, u_C(k)_, and u_G(k)_

respectively, providing a four-dimensional representation of the frequency spectrum of the character string. The quantity obtained by summing the squares of the Fourier transform of indicator sequences:

(4) S(k) = |*u*_*A*_(*k*)|^2 ^+ |*u*_*T*_(*k*)|^2 ^+ |*u*_*C*_(*k*)|^2 ^+ |*u*_*G*_(*k*)|^2^

is used as a measure of the total spectral content of DNA character string at frequency k [9,37,39,40,111].

Fourier transform of a nucleotide sequence was represented also by sum of pure sequences (Eq. 3) or by their product [15,109]. A single binary sequence was used by mapping genomic sequence into purine/pyrimidine representation [22], or into weak bond/strong bond representation [109]. Alternatively, mapping of DNA symbolic sequence into a set of quaternions could be utilized via the use of quaternionic Fourier transform [31].

A quartic mapping of genomic into numerical sequence of length N was performed by mapping each symbol to a number [17,37,111]:

(5) x(m) = au_A(m) _+ tu_T(m) _+ cu_C(m) _+ gu_G(m)_,   m = 0, 1, 2, ..., N-1

where a, t, c, and g are numerical values assigned to the characters A, T, C, and G, respectively.

We define the quartic map by ordering numbers of nucleotides with increasing frequency in the sequence AC017075.8 (corresponding to the orientation -) in chromosome 7, which is used for our case study:

(6) a = 4, t = 3, c = 2, g = 1

These values are in accordance with ordering of nucleotides with decreasing frequencies in the HOR region, and therefore they are biased in favor of A and T.

When using sequences from the Build 36.2 assembly (corresponding to the orientation +), the corresponding parametrization for quartic mapping is complement to (Eq. 6):

(7) t = 4, a = 3, g = 2, c = 1

In [113] the purine/pyrimidine and strong/weak bond properties of the four kinds of nucleotides were considered. The point (1,1) was used to represent nucleotide C corresponding to its pyrimidine and strong bond properties; the point (-1,1) to represent nucleotide G corresponding to its purine and strong bond properties; the point (-1,-1) to represent nucleotide A corresponding to its purine and weak bond properties; and the point (1,-1) to represent nucleotide T corresponding to its pyrimidine and weak bond properties. Then the vectors connecting the origin to the four points (1,1), (-1,1), (-1,-1) and (1,-1) have the rotational angles *π*/4, 3*π*/4, 5*π*/4,7*π*/4 with the x-axis and correspondingly the map defined [113]:

(8) a = 7, t = 5, c = 3, g = 1

These quartic map parameters are linearly related to parameters in (Eq. 6), b = 2b'-1 (b and b' stand for the corresponding nucleotides in (Eq. 8) and (Eq. 6), respectively).

In [37] the dimensionality of the frequency spectrum representation was reduced from four to three in a symmetric manner with respect to all four components. Three numerical sequences *ξ*_r_, *ξ*_g_, *ξ*_b _were defined from the corresponding coefficients (a_r_, t_r_, c_r_, g_r_), (a_g_, t_g_, c_g_, g_g_), (a_b_, t_b_, c_b_, g_b_) by considering the four three-dimensional vectors having magnitude equal to 1 and pointing to the four directions from the center to the vertices of regular tetrahedron:

(9) $$\xi_{\text{r}}=\frac{\sqrt{2}}{3}\left[2u_{T}(n)-u_{c}(n)-u_{G}(n)\right]$$

(10) $$\xi_{\text{g}}=\frac{\sqrt{6}}{3}\left[u_{c}(n)-u_{G}(n)\right]$$

(11) $$\xi_{\text{b}}=\frac{1}{3}\left[3u_{A}(n)-u_{T}(n)-u_{c}(n)-u_{G}(n)\right]$$

from which the DFTs are calculated.

In all computations the DFT was computed using Fast Fourier Transform (FFT) computer program [115] with the 1/$\sqrt{N}$ normalization.

A search for regions of higher order repeats in anonymous sequence, without prior knowledge on its structure, can be performed by sliding window analysis, similarly as used in Spectral Repeat Finder [16]. Once a region of HOR structure is detected, a more precise edge detection of HOR region can be determined by performing more precise local search using smaller step size.

## Abbreviations

HOR: Higher Order Repeat; KSA: Key String Algorithm; DFT: Discrete Fourier Transform.

## Authors' contributions

VP initiated the project and guided the whole work. VP and MR drafted the manuscript. NP, VP and IB implemented and developed the program for power spectrum with quartic mapping. NP, IB, MR, MG and NP were involved in computations and analysis of results. All authors participated in discussions and approved the final manuscript.
